# A four-herb topical formula attenuates psoriasis-like skin inflammation by suppressing IL-17-mediated inflammation and pathogenic γδT17 cell infiltration

**DOI:** 10.3389/fphar.2026.1957818

**Published:** 2026-10-28

**Authors:** Chun-Yao Cheng, Ping Chen, Dao-Syuan Wu, Hung-Rong Yen, Mei-Yun Chu, Shu-Cheng Tseng, Chia-Lin Lee, Chuan-Teng Liu, Ying-Chyi Song

**Affiliations:** 1 Department of Chinese Medicine, Taichung Tzu Chi Hospital, Taichung, Taiwan; 2 Graduate Institute of Integrated Medicine, College of Chinese Medicine, China Medical University, Taichung, Taiwan; 3 School of Chinese Medicine, College of Chinese Medicine, China Medical University, Taichung, Taiwan; 4 Research Center for Traditional Chinese Medicine, Department of Medical Research, China Medical University Hospital, Taichung, Taiwan; 5 Chinese Medicine Research Center, China Medical University, Taichung, Taiwan; 6 Department of Chinese Medicine, China Medical University Hospital, Taichung, Taiwan; 7 Office of Research and Development, Asia University, Taichung, Taiwan; 8 Department of Clinical Research Center of Integrated Medicine, Taichung Tzu Chi Hospital, Taichung, Taiwan; 9 Department of Cosmeceutics, China Medical University, Taichung, Taiwan; 10 Drug Development Research Unit, Research Center for Traditional Chinese Medicine, China Medical University Hospital, Taichung, Taiwan; 11 Department of Chinese Pharmaceutical Sciences and Chinese Medicine Resources, College of Chinese Medicine, China Medical University, Taichung, Taiwan

**Keywords:** IL-17, immunomodulation, psoriasis, Th17, traditional Chinese medicine, γδT17 cells

## Abstract

**Background:**

Psoriasis is a chronic immune-mediated inflammatory skin disease driven by dysregulation of IL-17-mediated immune responses. Current topical therapies, including corticosteroids, are limited by long-term side effects, creating demand for safer alternatives. DH-YH is a novel four-herb Traditional Chinese Medicine (TCM) formula derived from Duhuo Jisheng Tang, comprising *Angelica pubescens*, *Gentiana macrophylla*, *Taxillus chinensis*, and *Paeonia lactiflora*. Based on an initial clinical observation of improvement following topical DH-YH treatment in a patient with psoriasis, this study investigated its immunomodulatory effects in psoriasis-related models.

**Methods:**

The anti-inflammatory effects of DH-YH were assessed *in vitro* using IL-17A-stimulated human keratinocytes (HaCaT cells) and LPS-activated bone marrow-derived dendritic cells (BMDCs) and RAW264.7 macrophages. The effects on T-cell polarization were evaluated in murine splenic CD4^+^ T cells differentiated toward Th1, Th2, and Th17 lineages. *In vivo*, an imiquimod (IMQ)-induced psoriasis-like mouse model (BALB/c, n = 5/group) was used to evaluate topical DH-YH ointment. Skin histopathology (H&E staining) and multi-organ flow cytometric analysis (skin, spleen, and lymph nodes) were performed to assess epidermal thickness, inflammatory infiltration, and immune cell subset frequencies.

**Results:**

Topical DH-YH ointment markedly improved psoriatic lesions in a clinical case without hepatorenal toxicity. *In vitro*, DH-YH dose-dependently suppressed IL-8 secretion in IL-17A-stimulated HaCaT cells and inhibited TNF-α and IL-6 production in LPS-activated BMDCs. DH-YH also significantly reduced Th17 and Th2 cell differentiation without affecting cell viability. In the IMQ-induced mouse model, topical DH-YH significantly reduced epidermal thickness and dermal inflammatory cell infiltration. Flow cytometric analysis revealed that DH-YH specifically decreased the proportions of CD45^+^, CD3^+^, γδT, and γδT17 cells in lesional skin.

**Conclusion:**

DH-YH attenuates psoriasis-related inflammatory responses across multiple cellular and immune components, including keratinocyte inflammatory responses, BMDC cytokine production, T helper cell differentiation, and γδT17 cell accumulation.

## 1 Introduction

Psoriasis is a prevalent chronic, immune-mediated inflammatory skin disease affecting approximately 2%–3% of the global population (Sala et al., 2016). Clinically, it is characterized by well-demarcated erythematous plaques covered with silvery scales, resulting from excessive keratinocyte proliferation and abnormal differentiation (Nestle et al., 2009; Furue et al., 2020). Beyond the physical symptoms of pruritus and pain, psoriasis significantly impairs patients’ quality of life and is frequently associated with systemic comorbidities, including psoriatic arthritis and cardiovascular disease (Nestle et al., 2009). Current therapeutic strategies primarily include topical corticosteroids, phototherapy, and systemic biologics targeting specific cytokines (Mason et al., 2013; Strand et al., 2017; Armstrong and Read, 2020). However, long-term corticosteroid use can lead to skin atrophy and tachyphylaxis, while biologics, despite their high efficacy, are limited by high costs, the requirement for parenteral administration, and risks of serious infection (Menter et al., 2019). Consequently, there is a clear unmet clinical need for safer, more accessible, and effective topical agents for long-term psoriasis management.

The pathogenesis of psoriasis is driven by a complex interplay between innate and adaptive immune systems, centrally involving the IL-23/IL-17 axis (Cua et al., 2003). Upon activation by triggers such as mechanical stress or microbial stimuli, dendritic cells and macrophages produce IL-23, which promotes the expansion and activation of Th17 cells and γδT cells (Cai et al., 2011). These IL-17-producing cells secrete potent pro-inflammatory cytokines, notably IL-17A, which acts on keratinocytes to induce the production of chemokines (e.g., IL-8/CXCL8) and antimicrobial peptides (Mabuchi et al., 2011; Cai et al., 2013). This feed-forward inflammatory loop results in massive neutrophil infiltration and keratinocyte hyperproliferation (Nestle et al., 2009; Rendon and Schakel, 2019). Recent evidence has particularly highlighted the role of γδT17 cells as primary early responders in the skin, making the modulation of these cell subsets a focal point for therapeutic intervention (Pantelyushin et al., 2012; Ha et al., 2014).

Traditional Chinese Medicine (TCM) has a long history of treating inflammatory skin conditions (Weng et al., 2016; Lee et al., 2019; Lee et al., 2020; Lu et al., 2023). Du Huo Ji Sheng Tang (DJT, 獨活寄生湯), a classic formula recorded in the Tang Dynasty’s *Bei Ji Qian Jin Yao Fang* (備急千金要方), is traditionally used to “dispel wind-dampness” and “nourish blood to relieve pain.” While historically indicated for rheumatoid conditions, recent pharmacological studies and clinical observations suggest that DJT and its constituent herbs possess significant anti-inflammatory and immunomodulatory properties (Chen C. W. et al., 2011; He and Dai, 2011; Ma et al., 2019; Qin et al., 2022; Kaiyun et al., 2025). Based on accumulated clinical experience at Taichung Tzu Chi Hospital, a refined four-herb formula, DH-YH (comprising *Angelica pubescens*, *Gentiana macrophylla*, *Taxillus chinensis*, and *Paeonia lactiflora*), was developed to optimize these therapeutic effects for topical application in psoriasis. Given the cutaneous localization of psoriatic lesions, topical administration was selected to deliver DH-YH directly to the affected skin while minimizing systemic exposure; however, oral and topical administration were not directly compared in the present study.

Prompted by the clinical observation, we adopted a stepwise experimental approach to determine whether DH-YH modulates psoriasis-relevant inflammatory responses *in vitro* and whether the clinically used topical formulation attenuates psoriasiform inflammation *in vivo*. Despite the clinical potential of DH-YH, its specific regulatory effects on the psoriatic immune microenvironment remain to be fully elucidated. This study aimed to investigate the immunomodulatory effects of DH-YH using both *in vitro* and *in vivo* models. We examined the inhibitory effects of DH-YH on pro-inflammatory cytokine secretion in HaCaT keratinocytes, RAW264.7 macrophages, and BMDCs. Furthermore, using an IMQ-induced psoriasis-like mouse model (Liu et al., 2023; Lee et al., 2024), we evaluated the efficacy of topical DH-YH ointment in alleviating skin lesions and its ability to modulate the infiltration of pathogenic Th17 and γδT17 cells. Our findings provide a scientific rationale for the application of DH-YH as a novel topical treatment for psoriasis.

## 2 Materials and methods

### 2.1 Preparation and HPLC analysis of DH-YH water extract

DH-YH, a water extract containing four herbs—Duhuo (*A. pubescens*), Qinjiao (*G. macrophylla*), Sangjisheng (*T. chinensis*), and Baishao (*P. lactiflora*)—was obtained from Kang Li Biotechnology Co., Ltd. (Taichung, Taiwan). The botanical sources, taxonomic authorities, families, pharmacopoeial drug names, medicinal parts, and sourcing information are summarized in Table 1. Each of the four herbs was added in equal amounts (18.8 g each) to 1,000 mL of reverse-osmosis (RO) water, brought to a boil, and decocted for 30 min. The resulting decoction was centrifuged at 6,000 rpm for 10 min, filtered through a 0.45 μm membrane, and freeze-dried using a low-temperature vacuum concentrator to obtain a dry concentrated powder. The final stock solution was reconstituted to a concentration of 36.2 mg/mL. Independent batches of freshly prepared DH-YH decoction were used for clinical application, *in vitro* experiments, and animal studies. All batches were prepared using the same standardized extraction procedure described above.

**TABLE 1:** Botanical and pharmacopoeial information of the herbal materials used in DH-YH.

Herbal drug	Botanical source	Family	Pharmacopoeial drug name	Medicinal part	Source
Duhuo	*Angelica pubescens* Maxim. f. *biserrata* R.H.Shan and C.Q.Yuan	Apiaceae	Angelicae Pubescentis Radix	Dried root	Kang Li Biotechnology Co., Ltd., Taichung, Taiwan
Qinjiao	*Gentiana macrophylla* Pall.	Gentianaceae	Gentianae Macrophyllae Radix	Dried root	Kang Li Biotechnology Co., Ltd., Taichung, Taiwan
Sangjisheng	*Taxillus chinensis* (DC.) Danser	Loranthaceae	Taxilli Herba	Dried leafy stem and branch	Kang Li Biotechnology Co., Ltd., Taichung, Taiwan
Baishao	*Paeonia lactiflora* Pall.	Paeoniaceae	Paeoniae Radix Alba	Dried root	Kang Li Biotechnology Co., Ltd., Taichung, Taiwan

The major chemical constituents of the DH-YH extract were characterized by high-performance liquid chromatography (HPLC). Chromatographic analyses were performed using a Shimadzu HPLC system equipped with an LC-40D solvent delivery module, an SPD-40 UV–Vis detector, a DGU-403 degassing unit, and a CTO-40S column oven. Separation was achieved on a COSMOSIL® 5C18-MS-II column (4.6 × 250 mm, 5 μm; Nacalai Tesque, Kyoto, Japan) at a flow rate of 1.0 mL/min. The injection volume was 10 μL for both the DH-YH extract (36.2 mg/mL) and the reference standards. The column temperature was maintained at 30 °C, except for quercitrin analysis, which was performed at 25 °C. Authentic reference standards of osthol, gentiopicrin, quercitrin, and paeoniflorin were purchased from Qualiflex Co., Ltd. (Taichung, Taiwan). For osthol analysis, the mobile phase consisted of acetonitrile (MeCN) and water using the following gradient program: 52% MeCN for 25 min, followed by a linear increase to 100% MeCN over 10 min. UV detection was performed at 320 nm, and the osthol reference standard was prepared at 20 μg/mL. For gentiopicrin analysis, the mobile phase consisted of MeCN and 0.1% acetic acid in water under isocratic conditions (9% MeCN for 35 min). UV detection was performed at 254 nm, and the gentiopicrin reference standard was prepared at 1 mg/mL. For quercitrin analysis, the mobile phase consisted of MeCN and water using a gradient of 5%–10% MeCN over 5 min, followed by a linear increase to 100% MeCN over 25 min. UV detection was performed at 254 nm, and the quercitrin reference standard was prepared at 25 μg/mL. For paeoniflorin analysis, the mobile phase consisted of MeCN and 0.1% acetic acid in water under isocratic conditions (14% MeCN for 20 min). UV detection was performed at 230 nm, and the paeoniflorin reference standard was prepared at 1 mg/mL. The chromatographic profiles of the DH-YH extract were compared with those of the corresponding reference standards for the identification of the representative marker compounds (Figures 1A–D).

**FIGURE 1:**
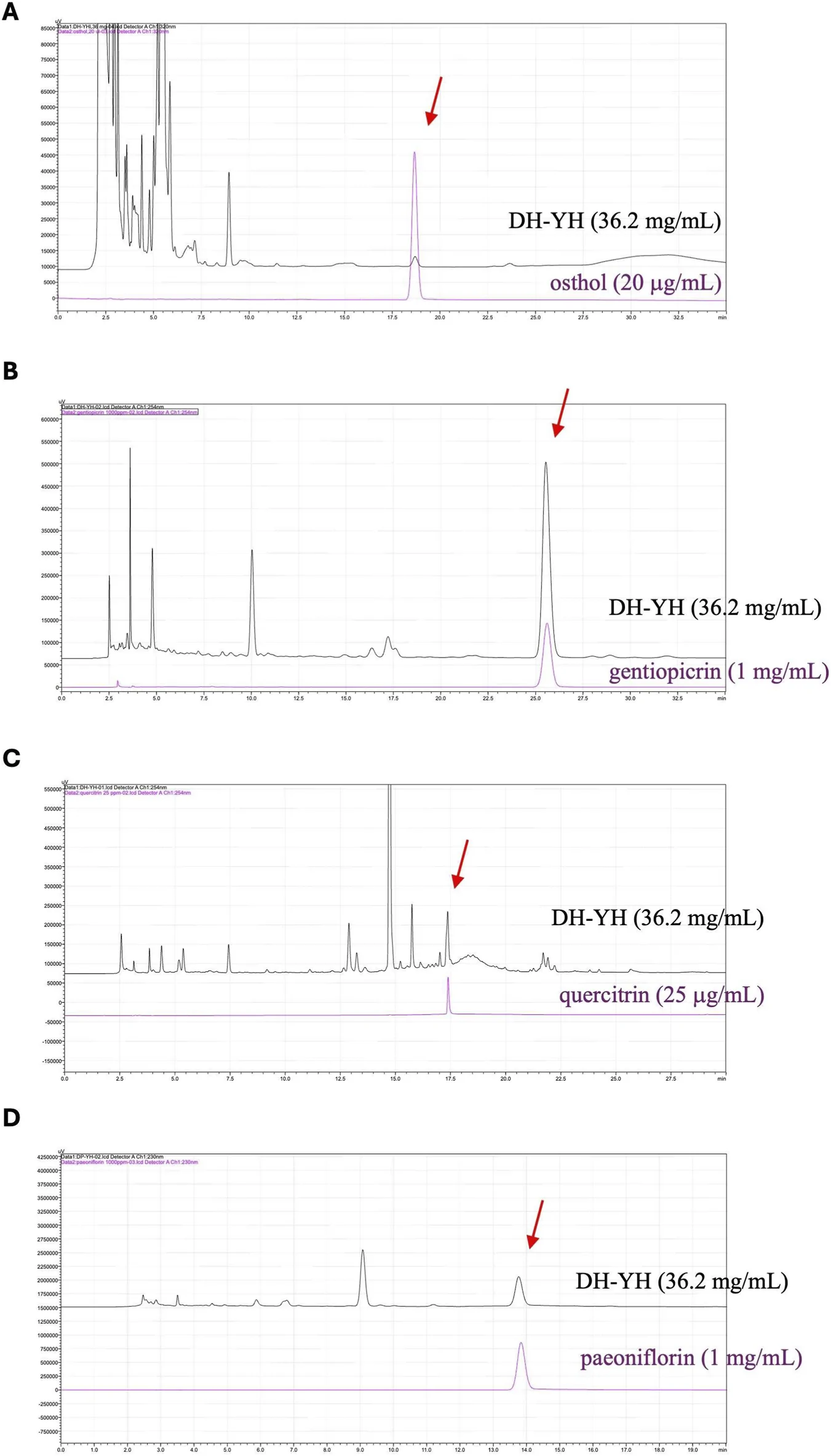
HPLC chromatographic identification of representative marker compounds in the DH-YH extract. Representative HPLC chromatograms of the DH-YH water extract (black) and authentic reference standards (pink). Arrows indicate the corresponding peaks of osthol **(A)**, gentiopicrin **(B)**, quercitrin **(C)**, and paeoniflorin **(D)** identified in the DH-YH extract by comparison with the respective reference standards.

### 2.2 Preparation of DH-YH ointment

The DH-YH ointment was formulated by adding 60 g of bitter almond oil (10% w/w) and 33 g of emulsifier (5% w/w) to approximately 600 mL of the final DH-YH decoction, followed by thorough mixing. A vehicle control ointment was prepared identically using RO water instead of the herbal decoction. For both clinical application and the animal experiments, the freshly prepared DH-YH decoction was used without further dilution to prepare the topical ointment.

### 2.3 Cell lines and culture conditions

The immortalized human keratinocyte cell line HaCaT was cultured in Dulbecco’s Modified Eagle Medium (DMEM; Gibco, Thermo Fisher Scientific, Waltham, MA, USA) supplemented with 5% fetal bovine serum (FBS; Sigma-Aldrich) and 1% penicillin-streptomycin, and maintained in a humidified 5% CO^2^ incubator at 37 °C. The murine macrophage cell line RAW264.7 was maintained in DMEM containing 10% FBS and 1% penicillin-streptomycin.

### 2.4 Generation of bone marrow-derived dendritic cells (BMDCs)

Bone marrow-derived dendritic cells (BMDCs) were generated from the femoral bone marrow of 8–12-week-old C57BL/6 mice as previously described by Song et al. (2020) and Song et al. (2022). Briefly, bone marrow cells were flushed from the medullary cavities using a 3 mL syringe with a 23-gauge needle in phosphate-buffered saline (PBS). After red blood cell lysis, cells were plated at 2 × 10^6^ cells per Petri dish in 10 mL of complete RPMI 1640 medium (Gibco) supplemented with 20 ng/mL recombinant mouse GM-CSF (PeproTech). On day 3, an additional 10 mL of fresh GM-CSF-containing medium was added. Non-adherent and loosely adherent BMDCs were harvested on day 6 and used for subsequent experiments.

### 2.5 *In vitro* stimulation and treatment

HaCaT cells (2 × 10^5^ cells/well) were stimulated with recombinant human IL-17A (100 ng/mL) PeproTech (USA) and treated with DH-YH water extract at concentrations ranging from 1.725% to 13.8% for 24 h. BMDCs and RAW264.7 macrophages were stimulated with lipopolysaccharide (LPS; 0.1 μg/mL) and treated with DH-YH water extract at concentrations ranging from 1.725% to 13.8% for 18 h. Cell culture supernatants were subsequently collected for cytokine (IL-6, IL-8, TNF-α) analysis by ELISA (BD Opt EIA™).

For T-cell polarization assays, purified mouse splenic CD4^+^ T cells were cultured under lineage-specific polarizing conditions as previously described (Song et al., 2022). For Th1 differentiation, plates were pre-coated with anti-CD3 antibody (5 μg/mL), and cells were cultured in the presence of soluble anti-CD28 antibody (1 μg/mL), IL-12 (30 ng/mL), IL-2 (5 ng/mL), and anti-IL-4 antibody (10 μg/mL) for 4 days. For Th17 differentiation, plates were pre-coated with anti-CD3 antibody (5 μg/mL), and cells were cultured with soluble anti-CD28 antibody (1 μg/mL), IL-6 (20 ng/mL), IL-23 (20 ng/mL), IL-1β (20 ng/mL), TGF-β (1.25 ng/mL), anti-IFN-γ antibody (10 μg/mL), and anti-IL-4 antibody (10 μg/mL) for 4 days. For Th2 differentiation, plates were pre-coated with anti-CD3 antibody (5 μg/mL), and cells were cultured with soluble anti-CD28 antibody (1 μg/mL), IL-4 (20 ng/mL), and anti-IFN-γ antibody (10 μg/mL) for 5 days. All antibodies and recombinant cytokines used for T-cell differentiation were purchased from BioLegend (San Diego, CA, USA). Following differentiation, cells were treated with DH-YH, dexamethasone (Dex; 5 μg/mL; Tai Yu Chemical and Pharmaceutical Co., Ltd.), or H_2_O vehicle control during differentiation for 16 h and then cells were stimulated with phorbol 12-myristate 13-acetate (PMA; 500 ng/mL, Sigma-Aldrich), ionomycin (500 ng/mL, Sigma-Aldrich), and GolgiStop (1 μL/mL, BD Biosciences) for an additional 5 h. Intracellular cytokine expression was subsequently analyzed using a FACSVerse flow cytometer (BD Biosciences).

### 2.6 Cytokine measurement and cell viability

Cytokine concentrations, including IL-6, IL-8, and TNF-α, in culture supernatants were quantified using commercial ELISA kits according to the manufacturers’ instructions (BD Biosciences, CA, USA). For intracellular cytokine staining, cells were fixed and permeabilized using the BD Cytofix/Cytoperm kit (BD Biosciences) according to the manufacturer’s protocol and subsequently stained with fluorochrome-conjugated antibodies against IL-17A, IL-4, and IFN-γ (BioLegend). Stained cells were analyzed using a FACSVerse flow cytometer (BD Biosciences), and data were processed using FlowJo software.

Cell viability of HaCaT cells was evaluated using the MTT assay (Sigma-Aldrich) (Lu et al., 2026). The viability of BMDCs, RAW264.7 macrophages, and differentiated Th1, Th2, and Th17 cells was assessed by propidium iodide (PI; Sigma-Aldrich) staining followed by flow cytometric analysis.

### 2.7 IMQ-induced psoriasis mouse model

BALB/c male mice (8–11 weeks old) (n = 5 per group) were randomly assigned to four groups: (1) untreated control (Con), (2) vehicle control (VC), (3) dexamethasone (Dex, 5 mg/kg), and (4) DH-YH ointment. The DH-YH ointment used in the animal study was prepared using the same undiluted DH-YH decoction as that used for the clinical formulation. On Day 0, the dorsal skin was shaved. From Day 1 to Day 6, each group received topical application of its respective treatment (150 μL/cm^2^). Thirty minutes later, all groups except the untreated control received topical 5% IMQ cream (62.5 mg/mouse/day) (Aldara®, Inova Pharmaceuticals/3M Healthcare) on the shaved dorsal skin. Animals were sacrificed on Day 7, and skin, lymph nodes, and spleen were collected. During the 6-day treatment period, the general activity and behavior of mice were monitored, and no overt differences were observed among the experimental groups. BALB/c male mice were obtained from the National Laboratory Animal Center (Taiwan). All animal experiments were performed in accordance with the guidelines of the Institutional Animal Care and Use Committee (CMUIACUC-2020-342-2).

### 2.8 Histological examination

Dorsal skin samples were fixed in 10% neutral buffered formalin, embedded in paraffin, sectioned at 3 μm, and stained with hematoxylin and eosin (H&E). Sections were visualized using a digital camera (Axiocam 105, Zeiss, Germany) mounted on a phase-contrast microscope (Zeiss) with Zen 2.3 software. Epidermal thickness and dermal inflammatory cell infiltration (cell count per field) were quantified from H&E-stained sections by a blinded observer.

### 2.9 Flow cytometric analysis of tissue immune cells

Single-cell suspensions were prepared from skin, draining lymph nodes, and spleens as previously described (Liu et al., 2023). Cells were stained with fluorochrome-conjugated antibodies against CD45 (BioLegend), CD3 (BD Biosciences), CD4 (BD Biosciences), and γδTCR (BioLegend) for surface marker analysis. For intracellular cytokine staining, cells were stimulated with PMA (500 ng/mL, Sigma-Aldrich), ionomycin (500 ng/mL, Sigma-Aldrich), and GolgiStop (1 μL/mL, BD Biosciences) for 5 h. Cells were then fixed and permeabilized using the Cytofix/Cytoperm kit (BD Biosciences) and stained with anti-IL-17A antibody (BioLegend). Flow cytometric data were acquired using a FACSVerse flow cytometer (BD Biosciences) and analyzed with FlowJo software.

### 2.10 Statistical analysis

Data are presented as mean ± standard deviation (SD). Statistical significance was evaluated by one-way analysis of variance (ANOVA) followed by Dunnett’s *post hoc* test for comparisons between experimental groups and the control. An unpaired Student’s t-test was used for comparisons between two groups. All analyses were performed using GraphPad Prism version 9.0 (GraphPad Software, San Diego, CA, USA). A *p*-value < 0.05 was considered statistically significant (*p < 0.05; **p < 0.01; ***p < 0.001; ****p < 0.0001).

## 3 Results

### 3.1 Clinical improvement of psoriatic lesions following topical DH-YH treatment in a single patient

The patient received topical DH-YH during routine clinical care by the treating Chinese medicine physician. The clinical course was subsequently documented after the observed improvement. The clinical case report was reviewed and approved by the Research Ethics Committee of Taichung Tzu Chi Hospital (No. REC-115-40). A 28-year-old male patient with a 3-year history of guttate psoriasis was treated with topical DH-YH ointment (15%, w/w) twice daily. The patient had previously received conventional treatments, including corticosteroids and antihistamines, with limited therapeutic benefit. Before DH-YH treatment, extensive erythematous papules and plaques with prominent scaling were observed on the trunk and extremities (Figure 2A). After 2 months of DH-YH treatment, marked clinical improvement was observed, including substantial reductions in erythema, scaling, and lesion size (Figure 2B). The psoriasis area and severity index (PASI) score decreased from 30.8 to 8.8. Although mild post-inflammatory hyperpigmentation remained in some areas, most active lesions had resolved. During follow-up, recurrent flare-ups associated with infection or stress were effectively controlled by reapplication of DH-YH ointment, and no local adverse reactions were observed.

**FIGURE 2:**
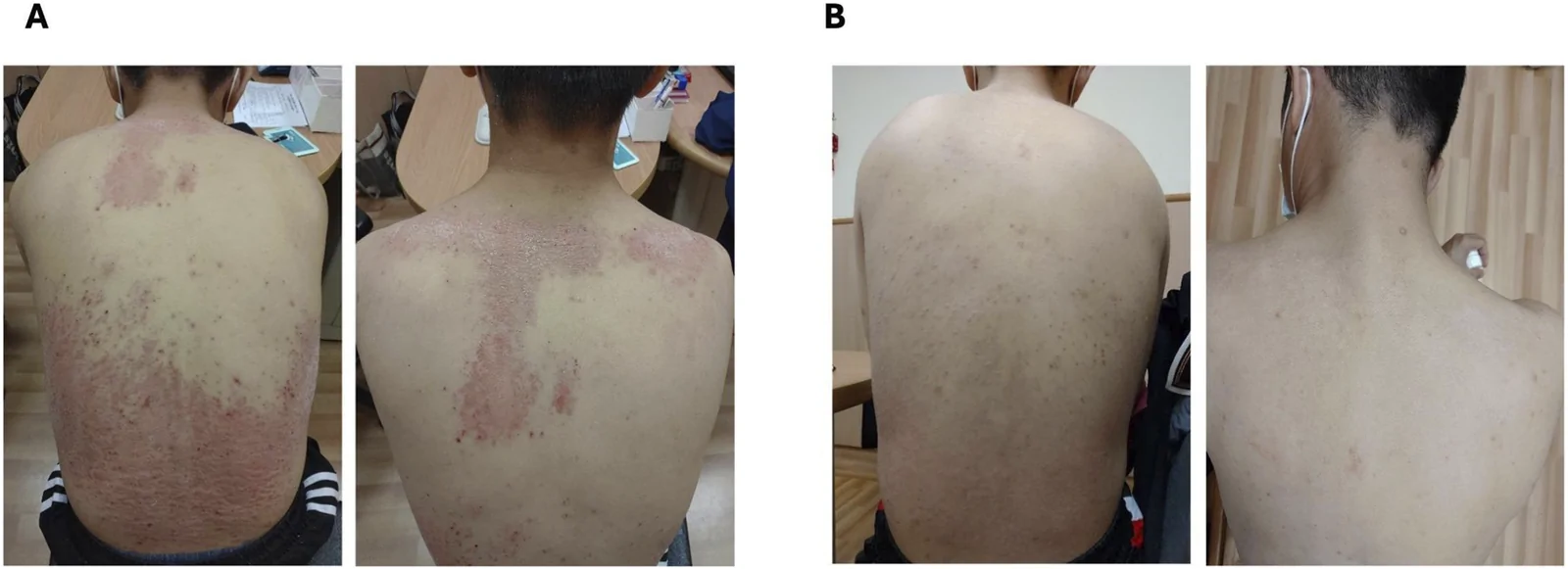
Clinical photographs of a patient with guttate psoriasis before and after topical DH-YH treatment. Representative clinical images of a patient with guttate psoriasis. **(A)** Baseline presentation showing multiple erythematous papules and plaques with prominent scaling on the trunk. **(B)** Clinical appearance after 2 months of twice-daily treatment with 15% (w/w) DH-YH ointment, showing marked reductions in erythema, scaling, and lesion severity. Written informed consent was obtained from the patient for publication of the clinical photographs.

Importantly, longitudinal blood biochemical analysis demonstrated that liver (AST, ALT) and kidney (BUN, creatinine) function markers remained within normal physiological ranges throughout the treatment period (Table 2). The measured liver and kidney function parameters remained within the reference ranges during the observation period, and no clinically apparent local adverse reactions were reported in this patient.

**TABLE 2:** Changes in liver and kidney function parameters before and after 2 months of topical DH-YH treatment in a patient with guttate psoriasis.

Assessment time point	AST (GOT) (IU/L)	ALT (GPT) (IU/L)	BUN (mg/dL)	CRE (mg/dL)	eGFR
Before treatment	13	7	8	0.6	170.51
After 2 months treatment	12	8	9	0.7	142.72

### 3.2 DH-YH inhibits inflammatory responses in keratinocytes and bone marrow-derived dendritic cells

To investigate the anti-inflammatory effects of DH-YH in psoriasis-related cell types, HaCaT keratinocytes were stimulated with IL-17A to mimic the inflammatory microenvironment of psoriatic skin. DH-YH treatment significantly reduced IL-8 secretion in IL-17A-stimulated HaCaT cells at concentrations of 6.9% and 13.8% (*p < 0.0001*) (Figure 3A), without affecting cell viability (Figure 3C). Notably, the inhibitory effect of DH-YH at 13.8% was greater than that observed in the dexamethasone-treated group (*p = 0.0587*). In contrast, DH-YH did not inhibit IL-6 production in IL-17A-stimulated HaCaT cells at any tested concentration (Figure 3B). We next examined the effects of DH-YH on innate immune cell activation. In LPS-stimulated BMDCs, DH-YH dose-dependently suppressed the production of TNF-α and IL-6 (both *p < 0.0001*) without affecting cell viability (Figures 3D–F). However, DH-YH did not significantly inhibit TNF-α or IL-6 secretion in LPS-stimulated RAW264.7 macrophages across all tested concentrations (Figures 3G–I). The lack of significant inhibition in RAW264.7 cells, in contrast to BMDCs, indicates that the anti-inflammatory effects of DH-YH were not uniformly observed across different myeloid cell types under the conditions tested. Collectively, these findings demonstrate that DH-YH exerts cell-type-dependent anti-inflammatory effects on psoriasis-relevant inflammatory responses.

**FIGURE 3:**
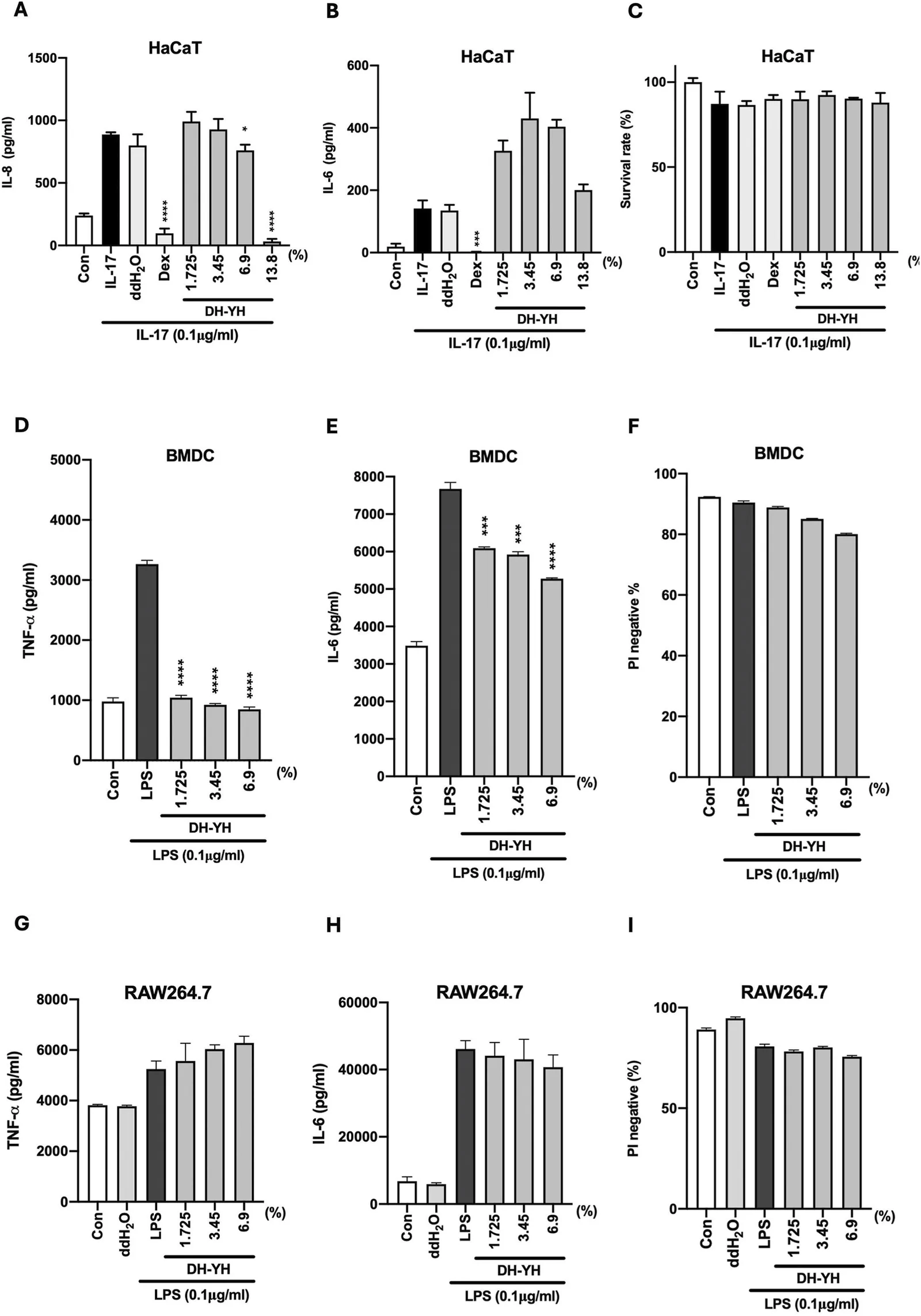
Effects of DH-YH on inflammatory cytokine production in HaCaT keratinocytes, BMDCs, and RAW264.7 macrophages. HaCaT keratinocytes, BMDCs, and RAW264.7 macrophages were treated with increasing concentrations of vehicle (ddH_2_O), DH-YH (1.725%–13.8%) or dexamethasone (Dex). **(A)** IL-8 and **(B)** IL-6 production in IL-17A-stimulated HaCaT cells. **(C)** HaCaT cell viability determined by MTT assay. **(D)** TNF-α and **(E)** IL-6 production in LPS-stimulated BMDCs. **(F)** BMDC viability determined by PI staining. **(G)** TNF-α and **(H)** IL-6 production in LPS-stimulated RAW264.7 macrophages. **(I)** RAW264.7 cell viability determined by PI staining. Data are presented as mean ± SD. *p < 0.05, ***p < 0.001, ****p < 0.0001 compared with the corresponding stimulated control group.

### 3.3 DH-YH suppresses Th17 and Th2 cell differentiation

Given that aberrant T helper cell polarization plays a critical role in psoriasis pathogenesis, we investigated the effects of DH-YH on the differentiation of murine CD4^+^ T cells under lineage-specific polarizing conditions. Under Th17-polarizing conditions, approximately 16.3% of CD4^+^ T cells in the vehicle control (H_2_O) group differentiated into IL-17A-producing Th17 cells. DH-YH treatment significantly and dose-dependently reduced the proportion of Th17 cells, with the highest concentration (13.8%) decreasing the frequency to 8.81% (*p < 0.001*). Notably, the inhibitory effect of DH-YH at 13.8% was significantly greater than that observed in the dexamethasone-treated group (Figure 4A). Similarly, DH-YH dose-dependently suppressed the differentiation of IL-4-producing Th2 cells. Compared with the vehicle control (H_2_O) group, treatment with 13.8% DH-YH reduced the proportion of Th2 cells by nearly fivefold (Figure 4B). In contrast, the differentiation of IFN-γ-producing Th1 cells was minimally affected across all tested concentrations of DH-YH (Figure 4C). Collectively, these findings indicate that DH-YH selectively suppresses Th17 and Th2 activation while exerting minimal effects on Th1, suggesting a preferential inhibitory effect on Th17- and Th2-associated immune responses. Cell viability remained unaffected at all tested DH-YH concentrations in Th1, Th2, and Th17 cultures. Representative flow cytometry plots for Th1, Th2, and Th17 differentiation are provided in Supplementary Figure S1.

**FIGURE 4:**
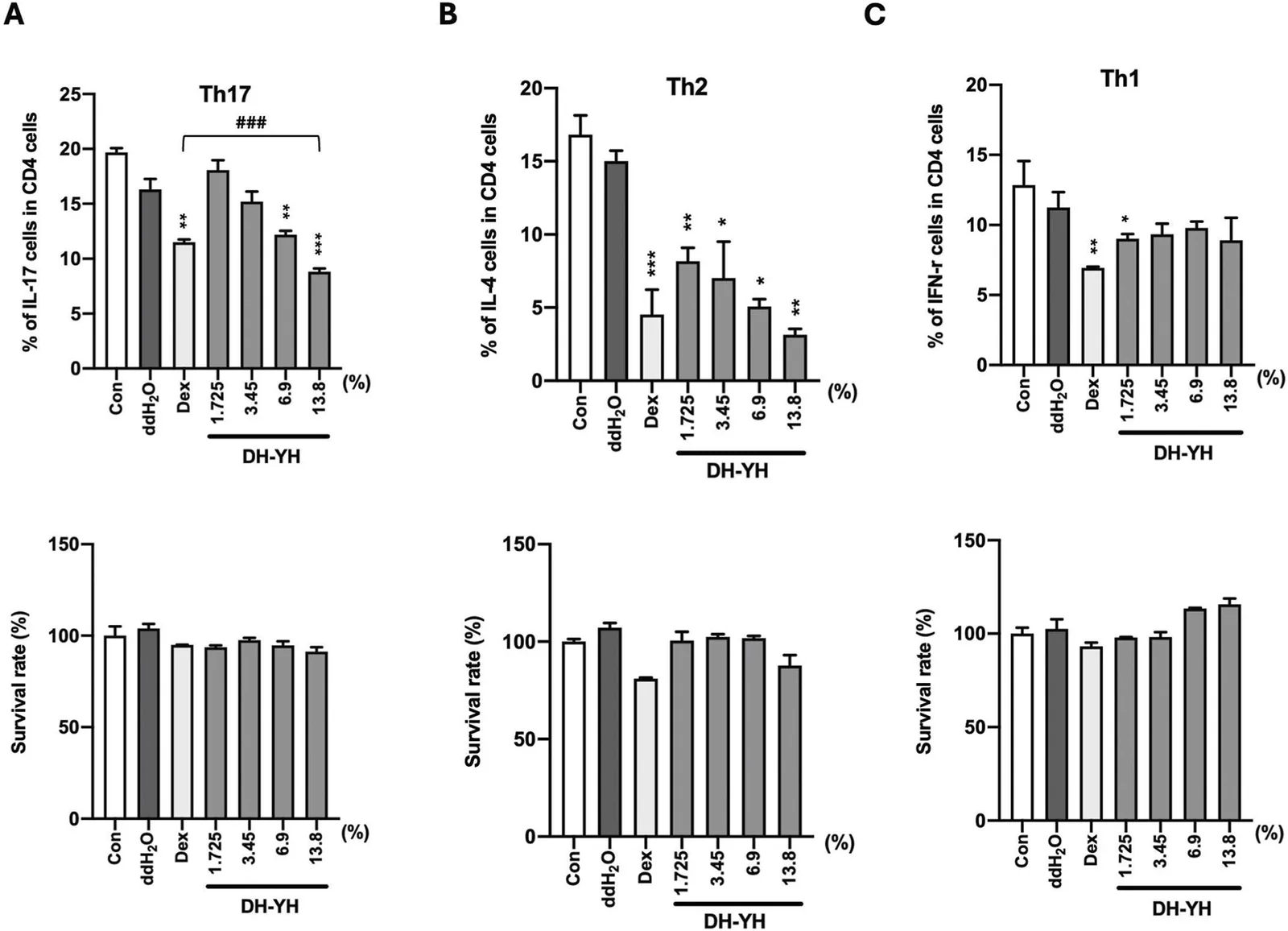
Effects of DH-YH on Th17, Th2, and Th1 cell differentiation. Purified mouse splenic CD4^+^ T cells were cultured under **(A)** Th17-, **(B)** Th2-, or **(C)** Th1-polarizing conditions. Following differentiation, cells were treated with vehicle (ddH_2_O), dexamethasone (Dex), or DH-YH (1.725%–13.8%) for 16 h and stimulated with PMA, ionomycin, and GolgiStop for an additional 5 h. Upper panels show the frequencies of IL-17A-producing Th17 cells **(A)**, IL-4-producing Th2 cells **(B)**, and IFN-γ-producing Th1 cells **(C)** determined by flow cytometry. Lower panels show the corresponding cell viability assessed by propidium iodide (PI) staining. Data are presented as mean ± SD. *p < 0.05, **p < 0.01, ***p < 0.001 versus the vehicle (ddH_2_O) group; ^*###*^ *p < 0.001* versus the Dex group.

### 3.4 Topical DH-YH attenuates IMQ-induced psoriasiform skin inflammation

To validate the anti-psoriatic effects of DH-YH *in vivo*, an IMQ-induced psoriasis-like mouse model was established (Figure 5A). Representative gross photographs of the dorsal skin from each experimental group are provided in Supplementary Figure S2. Gross examination did not reveal clear treatment-related differences among the IMQ-treated groups. Histological analysis was therefore used to further evaluate epidermal thickness and inflammatory cell infiltration. Histopathological examination of H&E-stained skin sections revealed marked epidermal hyperplasia in mice treated with vehicle ointment alone following six consecutive days of IMQ application, with a mean epidermal thickness of 205.04 μm (Figures 5B,C). In contrast, treatment with DH-YH ointment significantly reduced epidermal thickness to 117.14 μm (*p < 0.01* vs. vehicle ointment), indicating attenuation of IMQ-induced epidermal acanthosis. Consistent with these findings, quantitative analysis demonstrated that DH-YH ointment significantly reduced dermal inflammatory cell infiltration compared with vehicle ointment treatment (Figures 5D,E). The magnitude of inhibition was comparable to that observed in the dexamethasone-treated group. These findings suggest that topical DH-YH ointment attenuates IMQ-induced psoriasiform skin inflammation, as evidenced by reduced epidermal thickness and inflammatory cell infiltration.

**FIGURE 5:**
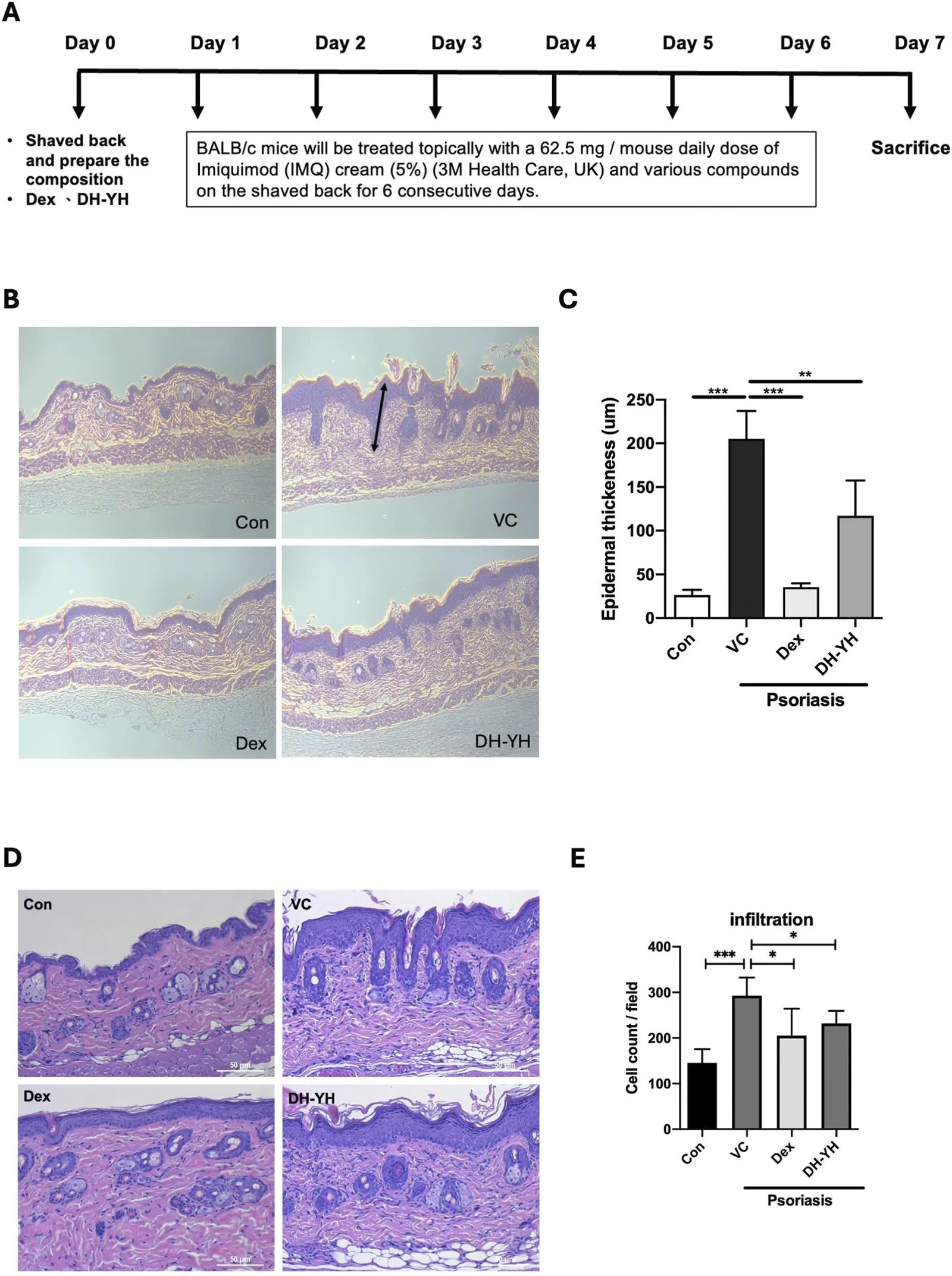
Effects of DH-YH ointment on IMQ-induced psoriasiform skin inflammation. Dorsal skin tissues from IMQ-induced psoriasis-like mice treated with vehicle ointment (VC), dexamethasone (Dex), or DH-YH ointment were analyzed by H&E staining. **(A)** Experimental design of the IMQ-induced psoriasis-like mouse model. BALB/c mice received topical treatment followed by daily application of 5% IMQ cream (62.5 mg/mouse) to the shaved dorsal skin for 6 consecutive days. Mice were euthanized on Day 7 for tissue collection and analysis. **(B)** Representative H&E-stained skin sections. Arrows indicate epidermal hyperplasia. **(C)** Quantification of epidermal thickness. **(D)** Representative higher-magnification H&E images showing dermal inflammatory cell infiltration. Scale bar = 50 μm. **(E)** Quantification of dermal inflammatory cell infiltration. Data are presented as mean ± SD (n = 5 mice per group). *p < 0.05, **p < 0.01, ***p < 0.001 versus the VC group.

### 3.5 DH-YH ointment reduces γδT and γδT17 cell accumulation in IMQ-induced psoriasiform skin

To further characterize the immunological changes associated with DH-YH treatment *in vivo*, immune cell populations in the skin, spleen, and draining lymph nodes of IMQ-treated mice were analyzed by flow cytometry (Figure 6). The flow cytometric gating strategy and representative plots for the analyzed T-cell populations in the skin, spleen, and draining lymph nodes are provided in Supplementary Figure S3. The most pronounced effects were observed in lesional skin. Compared with the vehicle ointment-treated group, DH-YH ointment significantly reduced the proportions of CD45^+^ leukocytes and CD3^+^ T cells in the skin (*p < 0.05* for both). Among the T-cell populations examined, DH-YH treatment was associated with significant reductions in both γδT cells and IL-17A-producing γδT17 cells within lesional skin (Figure 6A). Similar decreases in γδT and γδT17 cell frequencies were also observed in the spleen and draining lymph nodes following DH-YH treatment (Figures 6B,C). Collectively, these findings indicate that topical DH-YH ointment is associated with reduced leukocyte infiltration, particularly γδT and γδT17 cells, in IMQ-induced psoriasiform skin lesions, accompanied by corresponding changes in immune cell populations in peripheral lymphoid tissues.

**FIGURE 6:**
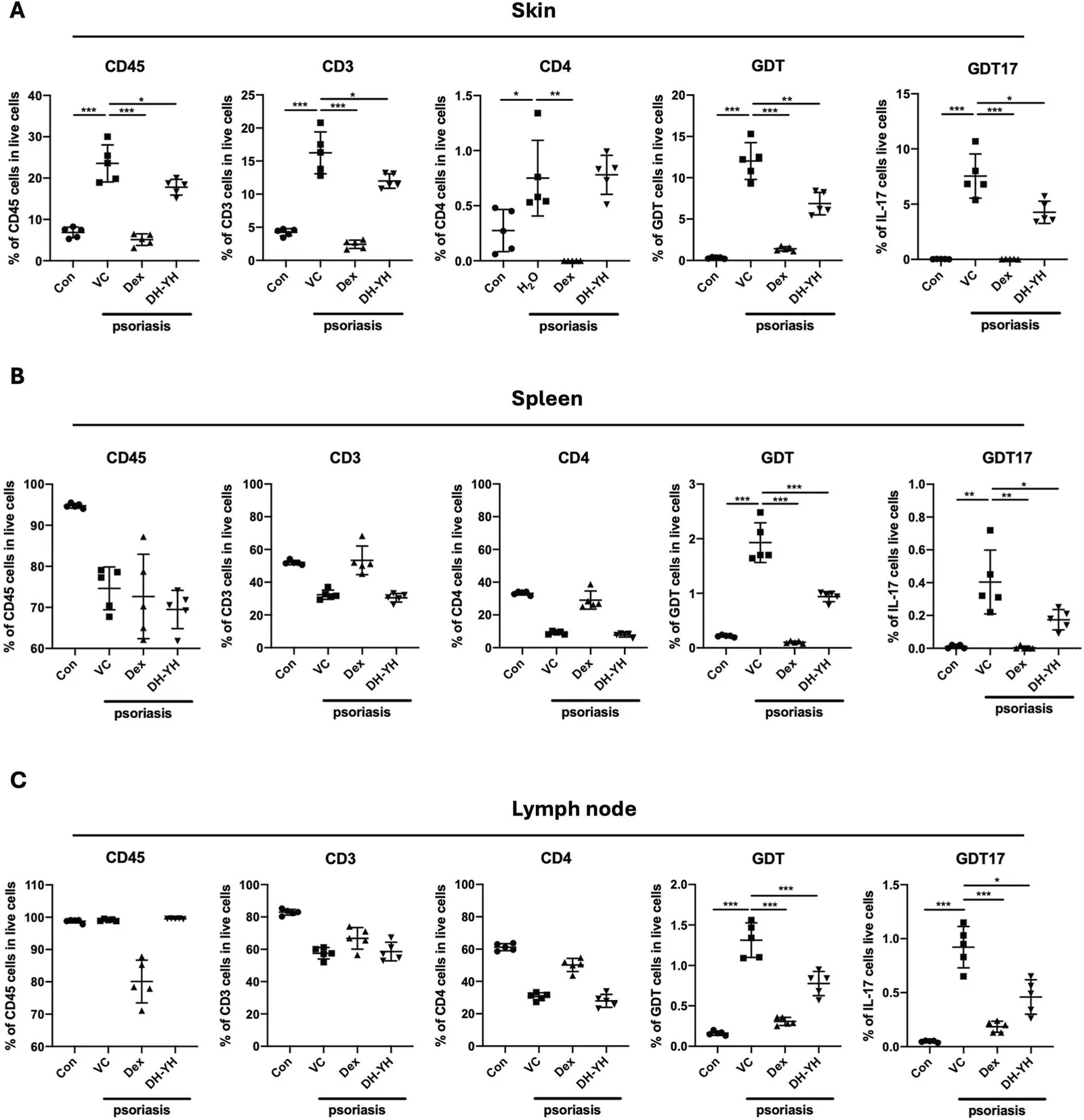
Effects of DH-YH ointment on immune cell populations in IMQ-induced psoriasis-like mice. Immune cell populations in the **(A)** skin, **(B)** spleen, and **(C)** draining lymph nodes were analyzed by flow cytometry following treatment with vehicle ointment (VC), dexamethasone (Dex), or DH-YH ointment. Frequencies of CD45^+^ leukocytes, CD3^+^ T cells, CD4^+^ T cells, γδT cells (GDT), and IL-17A-producing γδT17 cells (GDT17) are shown. Data are presented as mean ± SD (n = 5 mice per group). *p < 0.05, **p < 0.01, ***p < 0.001 versus the VC group.

## 4 Discussion

In this study, DH-YH exhibited immunomodulatory effects across several psoriasis-relevant cellular and immune components, including keratinocyte inflammatory responses, BMDC cytokine production, T helper cell differentiation, and γδT17 cell accumulation. These immunopharmacological findings collectively provide a scientific basis for the clinical improvement observed in our patient case and the therapeutic effects observed in the IMQ-induced mouse model (Gaffen et al., 2014). The clinical observation served as the rationale for the experimental investigation, while the cellular and animal studies provide complementary pharmacological evidence rather than direct mechanistic validation of the individual patient’s response.

The clinical improvement observed in the patient with guttate psoriasis provided the initial clinical observation that motivated the subsequent experimental investigation. The clinical recovery documented in our guttate psoriasis patient (Figure 2) aligns with prior reports emphasizing the responsiveness of this subtype to targeted immunomodulatory interventions (Jinna and Strober, 2016). Guttate psoriasis is characterized by acute T-cell activation and robust neutrophil recruitment driven by the IL-8/CXCR2 axis (Chiang et al., 2019; Padhi et al., 2026)—a pathway potentially related to the reduced IL-8 production observed following DH-YH treatment in our keratinocyte model.

A central finding of our *in vitro* experiments was the significant, dose-dependent inhibition of IL-8 (CXCL8) production in IL-17A-stimulated HaCaT cells. Notably, no inhibitory effect of DH-YH on IL-6 production was observed under the same conditions. This cytokine selectivity is therapeutically relevant: IL-8 is a potent chemoattractant for neutrophils and Th17 cells (Hammond et al., 1995), and keratinocyte-derived IL-8 plays a pivotal role in inflammatory cell recruitment to the epidermis, contributing to the formation of Munro’s microabscesses (Furue et al., 2020). Compared to other TCM formulas such as Yinxie I Formula, which has been reported to broadly suppress both IL-6 and IL-17 in a psoriasis model (Yanping et al., 2024), the differential cytokine response to DH-YH suggests distinct effects on individual inflammatory mediators. In addition, Smilax glabra Roxb. extract has been reported to alleviate IMQ-induced psoriasis-like lesions through TOP2A inhibition and AhR activation, further demonstrating the diverse immunopharmacological effects of herbal interventions in psoriasis (Wu et al., 2025). The reduction of dermal inflammatory cell infiltration observed histologically in DH-YH-treated mice (Figure 5) is consistent with the anti-inflammatory effects of DH-YH observed *in vitro* (Liu et al., 2025).

The differential responses of BMDCs and RAW264.7 macrophages suggest cell-type-dependent effects of DH-YH. Previous studies have demonstrated distinct signaling characteristics in dendritic cells and macrophages; however, these signaling pathways were not directly examined in the present study, and the molecular basis for the differential responses remains to be determined. BMDCs exhibit high responsiveness to NF-κB-dependent transcriptional regulation during maturation (Chen G. et al., 2011). Paeoniflorin, an active constituent of *P. lactiflora* (a key component of DH-YH), has been reported to inhibit IL-6 production in BMDCs and suppress Th17 differentiation through inhibition of the IKK/NF-κB and MAPK signaling pathways (Zhang et al., 2017). In contrast, LPS-stimulated RAW264.7 macrophages activate multiple redundant downstream pathways, including both MyD88-and TRIF-dependent cascades (Cheng et al., 2015), although whether these signaling characteristics contribute to the differential response to DH-YH remains unknown. Although RAW264.7 macrophages were included for cell-type comparison *in vitro*, macrophage populations were not specifically characterized *in vivo*; therefore, potential effects of DH-YH on tissue macrophages cannot be excluded.

The suppression of both Th17 and Th2 cell differentiation by DH-YH *in vitro* is noteworthy. Although psoriasis is canonically classified as a Th17-driven disease, and atopic dermatitis (AD) as a Th2-dominant inflammatory disorder, emerging evidence suggests that these conditions exist on an immunological continuum, with dynamic plasticity between Th17 and Th2 immune states (Nestle et al., 2009; Raychaudhuri, 2013; Weidinger and Novak, 2016; Guttman-Yassky and Krueger, 2017). The ability of DH-YH to simultaneously dampen both lineages may confer broader applicability across the spectrum of inflammatory skin diseases. In addition to classical Th17 cells, dermal γδT17 cells represent another critical source of pathogenic IL-17 in psoriatic skin (Cai et al., 2011). Our flow cytometric data demonstrated that DH-YH specifically and significantly reduced γδT and γδT17 populations in lesional skin, spleen, and lymph nodes (Figure 6), suggesting systemic modulation of this pathogenic axis rather than merely local anti-inflammatory effects.

Several limitations of this study warrant consideration. First, although the IMQ-induced model effectively recapitulates the acute inflammatory signature and the IL-23/IL-17/γδT17 axis of psoriasis, it does not fully replicate the chronic relapsing-remitting course or the complex genetic background of human disease. Furthermore, although IL-23 production was examined in LPS-stimulated BMDC supernatants, its concentration was below the reliable detection range of the assay under the present experimental conditions; therefore, whether DH-YH modulates dendritic cell-derived IL-23 remains to be determined. Second, DH-YH suppressed IL-8 but not IL-6 production in IL-17A-stimulated keratinocytes, indicating differential effects on keratinocyte-derived inflammatory mediators. The biological significance and underlying mechanisms of this differential response remain to be determined. Third, the clinical observation presented in this study represents a single case and should not be interpreted as evidence of clinical efficacy. Rather, it served as an initial observation that prompted subsequent experimental investigation of DH-YH. Larger prospective and controlled clinical studies are required. Fourth, the precise phytochemical constituents responsible for the differential immunomodulation of BMDCs versus RAW264.7 macrophages remain to be identified, and further pharmacological profiling of DH-YH’s active components and their synergistic interactions is warranted. Fifth, although independently prepared DH-YH batches produced directionally consistent pharmacological effects across the experimental settings, formal quantitative batch-to-batch chemical equivalence was not performed. Future development will require predefined chromatographic quality-control criteria and quantitative standardization of representative marker compounds.

In conclusion, DH-YH attenuates psoriasis-like inflammation through coordinated suppression of keratinocyte IL-8 production, BMDC-mediated innate activation, Th17/Th2 cell polarization, and pathogenic γδT17 cell infiltration into psoriatic skin. These multi-layered immunomodulatory effects, combined with a favorable clinical safety profile, support the further development of DH-YH as an effective and safe topical option for psoriasis management.

## 5 Conclusion

The present study demonstrates that DH-YH attenuates psoriasis-like skin inflammation and modulates several psoriasis-relevant immune responses. The single clinical observation provides an initial translational context for these experimental findings but does not establish clinical efficacy or safety. Future larger-scale clinical investigations are needed to establish standardized therapeutic protocols and to fully characterize the active constituents responsible for these immunomodulatory activities.

## Data Availability

The raw data supporting the conclusions of this article will be made available by the authors, without undue reservation.
