# Supplementary material for: A four-herb topical formula attenuates psoriasis-like skin inflammation by suppressing IL-17-mediated inflammation and pathogenic γδT17 cell infiltration
Source: Front Pharmacol. 2026 Oct 28;17:1957818. doi: 10.3389/fphar.2026.1957818 (PMC13751984; doi:10.3389/fphar.2026.1957818)
Supplement: Supplementary file 1 [file DataSheet1.pdf]

Supplementary Figure S1

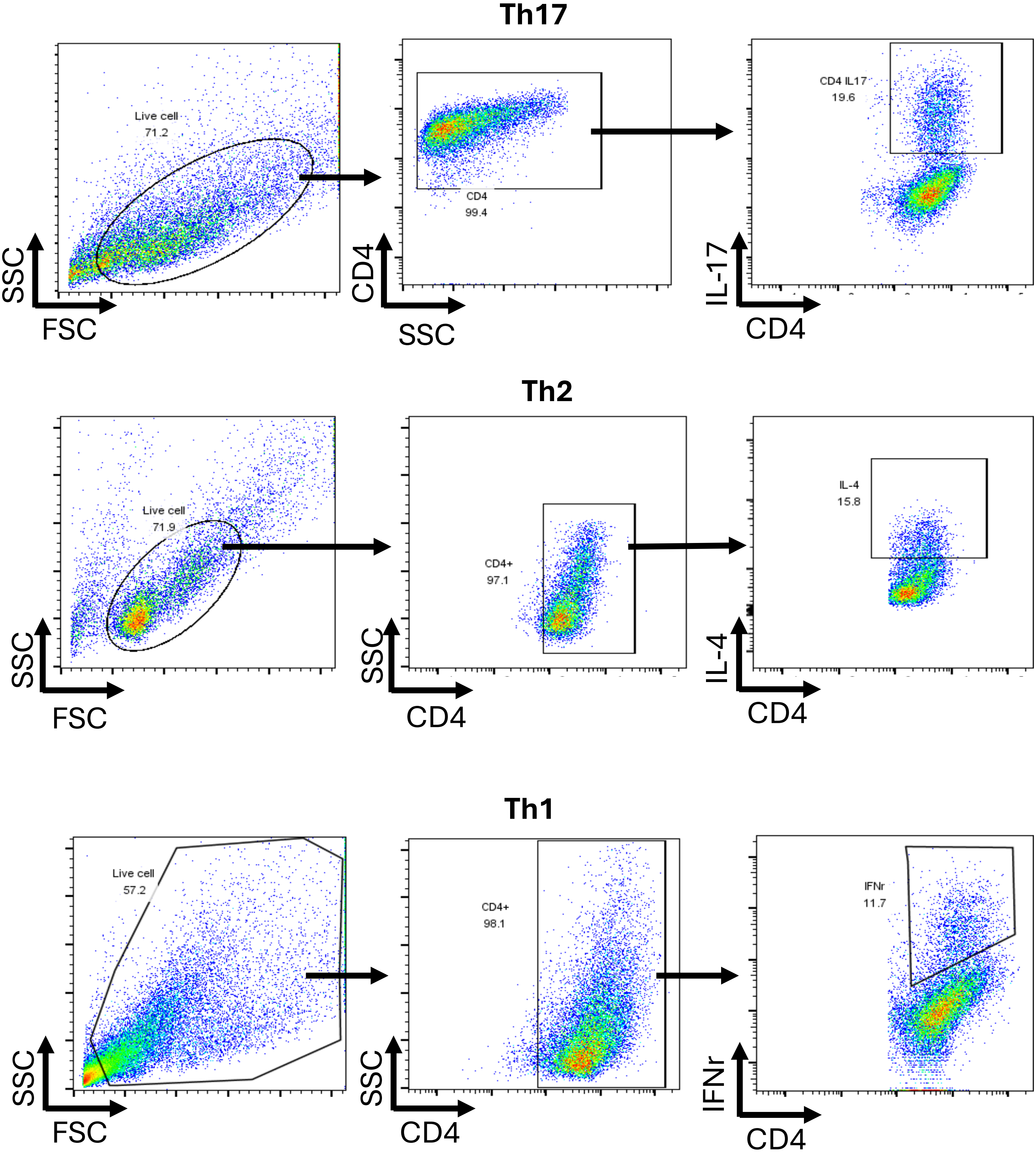

Supplementary Figure S1. Representative flow cytometry plots of in vitro T helper cell differentiation.

Representative gating and flow cytometry plots for Th1, Th17, and Th2 cells are shown. Following the indicated polarization conditions, T-cell subsets were identified based on intracellular cytokine expression in CD4+ T cells: IFN-gamma for Th1 cells, IL-17A for Th17 cells, and IL-4 for Th2 cells.

Supplementary Figure S2

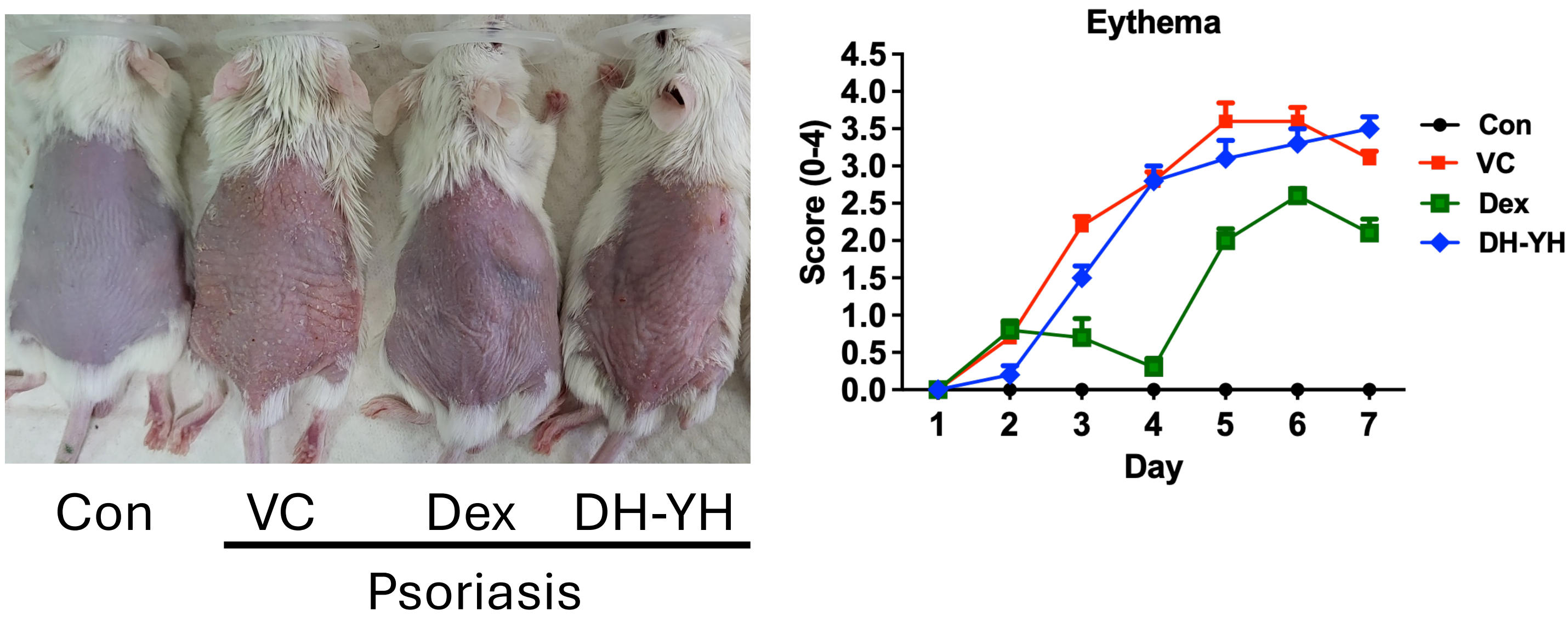

Supplementary Figure S2. Representative photographs of dorsal skin lesions in the IMQ-induced psoriasis-like mouse model.

Representative gross photographs of the dorsal skin from the control (Con), vehicle control (VC), dexamethasone-treated (Dex), and DH-YH-treated groups are shown following the experimental treatment period. These images provide gross morphological documentation corresponding to the histopathological and quantitative analyses presented in Figure 5.

Supplementary Figure S3

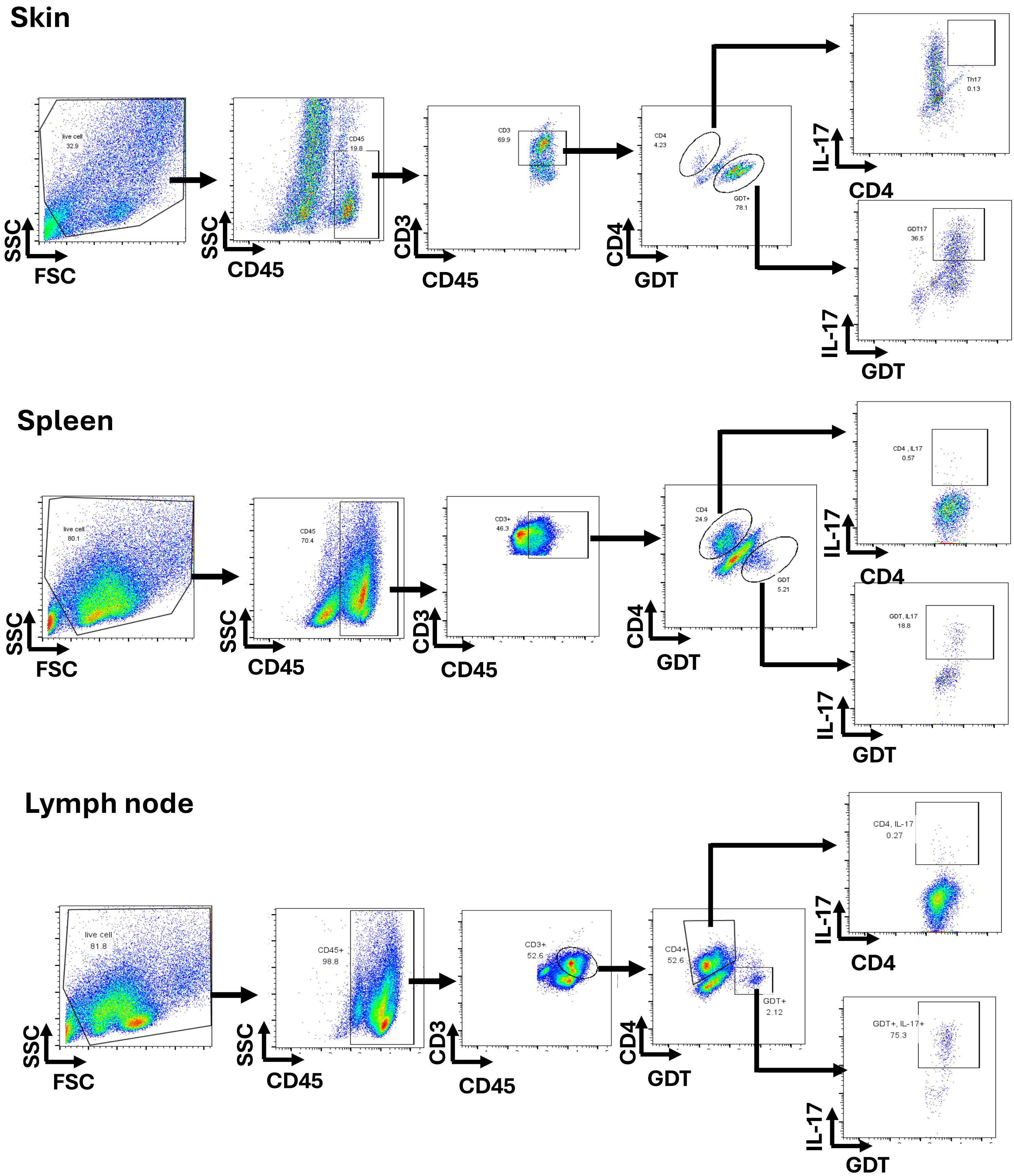

Supplementary Figure S3. Flow cytometric gating strategy and representative plots of T-cell populations in the skin, spleen, and draining lymph nodes.

Representative flow cytometric gating strategies and plots used for the analysis of immune cell populations in the skin, spleen, and draining lymph nodes of mice from the IMQ-induced psoriasis-like model are shown. Live cells were sequentially analyzed for CD45+ leukocytes and T-cell populations, including CD3+ T cells, CD4+ T cells, γδT cells (GDT), and IL-17A-producing γδT cells, as applicable to the respective analyses.
